# Metabolic, Molecular, and Behavioral Effects of Western Diet in Serotonin Transporter-Deficient Mice: Rescue by Heterozygosity?

**DOI:** 10.3389/fnins.2020.00024

**Published:** 2020-02-18

**Authors:** Ekaterina Veniaminova, Raymond Cespuglio, Irina Chernukha, Angelika G. Schmitt-Boehrer, Sergey Morozov, Allan V. Kalueff, Oxana Kuznetsova, Daniel C. Anthony, Klaus-Peter Lesch, Tatyana Strekalova

**Affiliations:** ^1^Department of Psychiatry and Neuropsychology, School for Mental Health and Neuroscience, Maastricht University, Maastricht, Netherlands; ^2^Laboratory of Psychiatric Neurobiology, Institute of Molecular Medicine, Sechenov First Moscow State Medical University, Moscow, Russia; ^3^Faculty of Medicine, Neuroscience Research Center of Lyon, C. Bernard University Lyon 1, Lyon, France; ^4^V.M. Gorbatov Federal Research Center for Food Systems of RAS, Moscow, Russia; ^5^Division of Molecular Psychiatry, Center of Mental Health, University of Würzburg, Würzburg, Germany; ^6^Institute of General Pathology and Pathophysiology, Moscow, Russia; ^7^School of Pharmacy, Southwest University, Chongqing, China; ^8^Institute of Translational Biomedicine, St. Petersburg State University, St. Petersburg, Russia; ^9^Ural Federal University, Ekaterinburg, Russia; ^10^Department of Pharmacology, Oxford University, Oxford, United Kingdom

**Keywords:** *Sert*-deficient mice, Western diet, aging, glucose tolerance, Toll-like receptor 4 (TLR4), serotonin receptors, obesity, heterosis

## Abstract

Reduced function of the serotonin transporter (SERT) is associated with increased susceptibility to anxiety and depression and with type-2 diabetes, which is especially true in older women. Preference for a “Western diet” (WD), enriched with saturated fat, cholesterol, and sugars, may aggravate these conditions. In previous studies, decreased glucose tolerance, central and peripheral inflammation, dyslipidemia, emotional, cognitive, and social abnormalities were reported in WD-fed young female mice. We investigated the metabolic, molecular, and behavioral changes associated with a 3-week-long dietary regime of either the WD or control diet in 12-month-old female mice with three different *Sert* genotypes: homozygous (*Slc6a4*) gene knockout (*Sert*^−/−^: KO), heterozygous (*Sert*^+/−^: HET), or wild-type mice (*Sert*^+/+^: WT). In the WT-WD and KO-WD groups, but not in HET-WD-fed mice, most of changes induced by the WD paralleled those found in the younger mice, including brain overexpression of inflammatory marker Toll-like receptor 4 (*Tlr4*) and impaired hippocampus-dependent performance in the marble test. However, the 12-month-old female mice became obese. Control diet KO mice exhibited impaired hippocampal-dependent behaviors, increased brain expression of the serotonin receptors *Htr2c* and *Htr1b*, as well as increased *Tlr4* and mitochondrial regulator, peroxisome proliferator-activated receptor gamma-coactivator-1a (*Ppargc1a*). Paradoxically, these, and other changes, were reversed in KO-WD mutants, suggesting a complex interplay between *Sert* deficiency and metabolic factors as well as potential compensatory molecular mechanisms that might be disrupted by the WD exposure. Most, but not all, of the changes in gene expression in the brain and liver of KO mice were not exhibited by the HET mice fed with either diet. Some of the WD-induced changes were similar in the KO-WD and HET-WD-fed mice, but the latter displayed a “rescued” phenotype in terms of diet-induced abnormalities in glucose tolerance, neuroinflammation, and hippocampus-dependent performance. Thus, complete versus partial *Sert* inactivation in aged mice results in distinct metabolic, molecular, and behavioral consequences in response to the WD. Our findings show that *Sert*^+/−^ mice are resilient to certain environmental challenges and support the concept of heterosis as evolutionary adaptive mechanism.

## Introduction

Serotonin transporter (SERT), a key element of serotonergic neurotransmission (Collier et al., 1996; Murphy et al., 2004), is also involved in the regulation of metabolic processes (Stuart and Baune, 2012; Giannaccini et al., 2013; Pomytkin et al., 2015, 2018). In humans, a variant of the upstream regulatory region of the SERT (*SLC6A4*) gene, the so-called short (s) allele, in comparison with long (l) allele is associated with lower SERT activity and stressed-related vulnerability to anxiety and depression (Lesch et al., 1996; Greenberg et al., 2000; Caspi et al., 2010), and also with higher body mass index (BMI) (Sookoian et al., 2007; Fuemmeler et al., 2008) and incidence of type-2 diabetes (Iordanidou et al., 2010), which are typical for the female sex and aging (Kautzky-Willer et al., 2016; Khabazkhoob et al., 2017; Batsis and Zagaria, 2018).

Individuals with metabolic syndrome and obesity display decreased SERT expression in the brain and periphery (Giannaccini et al., 2013; Nam et al., 2018). Excessive intake of a hypercaloric diet, enriched with saturated fat and sugars, was shown to suppress the binding of hypothalamic SERT in obese subjects and in insulin-resistant subjects that is independent of body weight gain (Koopman et al., 2013; Versteeg et al., 2017). Thus, diminished SERT activity is believed to underlie negative changes associated with metabolic syndrome (Stuart and Baune, 2012) and in turn, metabolic abnormalities resulting in reduced SERT function that can contribute to emotional disturbances (Pomytkin et al., 2015, 2018).

Animal studies support the observations made in humans concerning the relationship between SERT deficiency and diabetes-like metabolic changes. Sert-deficient mice (*Sert*^−/−^: KO) were reported to display decreased glucose tolerance, increased deposition of white adipose tissue that increases with aging, and late-onset obesity; these changes were particularly marked in females (Murphy and Lesch, 2008; Üçeyler et al., 2010; Chen et al., 2012; Zha et al., 2017).

Aging is well known to be associated with compromised metabolic function (Boemi et al., 2016) and changes in serotonergic regulation (Rodríguez et al., 2012). Abnormal distribution of fat in the elderly increases the risk or exacerbates the negative effects of obesity on metabolic function, including a decline in insulin sensitivity and glucose tolerance (Morita et al., 2006; Karakelides et al., 2010). In humans, each decade results in a 10% decrease in the density of SERT-binding sites in the brain stem and thalamus (Yamamoto et al., 2002); this decrease is also found in monkeys and mice (Kakiuchi et al., 2001; Herrera-Pérez et al., 2013). Aging also results in a decrease in circulating serotonin and alterations in the densities of the brain serotonin receptors 5-HT1A, 5-HT2A, and 5-HT1B (Meltzer et al., 1998; Matuskey et al., 2012) that are more profound in women (Meltzer et al., 1998).

Despite the evidence of a relationship between genetic SERT deficiency and diabetes-like metabolic conditions, little is known about the underlining molecular mechanisms. In the human population, the combination of genetic SERT deficiency with an increased intake of the Western diet (WD) during aging is widespread, but the interactions are difficult to explore. Mutant animals provide an opportunity to model the interactions between genotype, diet, and age. Most studies addressing the impact of decreased SERT function on metabolic regulation during hypercaloric dietary challenge were performed with young *Sert*-deficient mice. For example, Chen et al. (2012) reported the presence of elevated fasting glucose levels, impaired glucose tolerance, and insulin resistance in *Sert^−/−^* animals exposed to a high-fat diet for 3 months in male mice. *Sert*^−/−^ female, but not male, rats demonstrated increased abdominal fat when fed either standard chow or a diet high in fat and sugar content (Homberg et al., 2010).

Concerning the effects of the Western diet and aging on the metabolic characteristics of *Sert* heterozygous (*Sert*^+/−^: HET) animals, very limited literature is available. It was reported that diet-induced metabolic changes in young SERT heterozygous mice are intermediate in their magnitude, showing impaired glucose tolerance and insulin resistance, with respect to the changes in the wild-type and knockout phenotypes (Chen et al., 2012). However, HET mice are considered to be qualitatively distinct from *Sert*^−/−^ animals and closer mimic the short allele human condition, displaying allelic variation of SERT function. Substantial differences between two genotypes were described in a maternal separation and other stress models, in which the HPA axis was suggested not to affect in the same way in HET and KO animals (Jiang et al., 2009; van der Doelen et al., 2014). In a prenatal stress model, HET mice demonstrated signs of superior stress resilience compared to WT, displaying reduced scores of anxiety-like behavior and improved cognitive performance (van den Hove et al., 2011), while other studies showed increased stress reactivity in KO (Wellman et al., 2007; Bearer et al., 2018).

In the present study, we sought to investigate metabolic, molecular, and behavioral changes induced by the WD in aged mice with complete or partial genetic SERT deficit. We employed a previously validated model that involves feeding mice with the WD for 3 weeks, and we evaluated metabolic and neurobiological hallmarks of the WD-induced syndrome *in vitro* and *ex vivo* assays (Strekalova et al., 2015, 2016; Veniaminova et al., 2016, 2017, 2020). In this model, impaired glucose tolerance, increases in cholesterol and leptin blood levels, brain and liver overexpression of Toll-like receptor 4 (*Tlr4*), decreased expression of mitochondrial markers peroxisome proliferator-activated receptor gamma coactivator 1 (*Ppargc1*) a and b, and decreased *Sert* expression in the brain are all features. These molecular changes are accompanied by depressive- and anxiety-like behaviors, signs of impulsivity, lowered sociability, and cognitive deficits (Strekalova et al., 2015, 2016; Veniaminova et al., 2016, 2017, 2020). Here, in view of the changes observed in 5-HT receptor densities in aging and in those with SERT deficiency, we also studied gene expression of serotonin receptors *Htr1a*, *Htr1b*, and *Htr2a* in the brain. The expression of the serotonin receptors *Htr2c* and *Htr6* were also examined owing to their known role in the regulation of emotionality and metabolic function (Bickerdike, 2003; Millan, 2005; Heal et al., 2008; Wesołowska, 2010).

## Materials and Methods

### Animals

Experiments were performed using 12-month-old homozygous *Sert*^−/−^ and heterozygous *Sert*^+/−^ female mice and wild-type littermates born from heterozygous mutants at the 10th generation (F10) of backcrossing with C57BL/6J mice; all genotypes were confirmed by PCR. Mice were housed three to four per cage during the study, under a reversed 12-h light–dark cycle (lights on: 21:00 h) with food and water *ad libitum* and under controllable laboratory conditions (22 ± 1°C, 55% humidity). Laboratory housing conditions and experimental procedures were set up and maintained in accordance with the European Communities Council Directive for the care and use of laboratory animals (2010/63/EU) and approved by the local ethics committees of C. Bernard University and MSMU (#11–18).

### Study Design and Diets

Mice were fed with a standard laboratory diet (control diet, CD) with an energy content of 3.8 kcal/g, 4.3% of fat (1.3% of saturated fat) (D18071801, Research Diet Inc., New Brunswick, NJ, United States) or with a diet containing 0.2% of cholesterol, 21.3% of fat (10.5% of saturated fat), and an energy content of 4.6 kcal/g, Western diet (D11012302, Research Diet Inc., New Brunswick, NJ, United States) for 3 weeks as described elsewhere (Strekalova et al., 2015, 2016; Veniaminova et al., 2017). The content of the nutrients in calories and weight and the ingredients are indicated in Supplementary Table S1. Body weight and intake of diet and water were monitored weekly (on days 1, 8, 15, and 21). Daily intake of calories and water was normalized to body weight.

**FIGURE 1:**
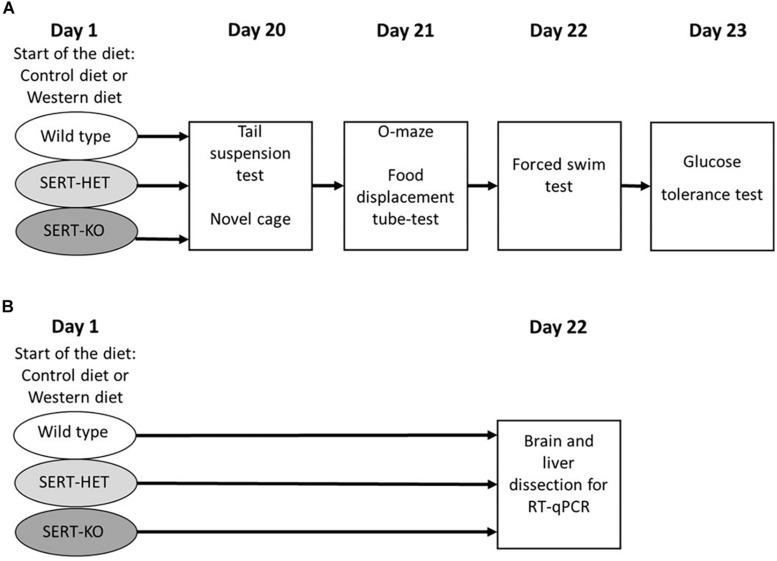
Experimental design. **(A)** Study of the effects of 3-week “Western diet” (WD) feeding of wild-type (WT), heterozygous (HET), and knockout (KO) mice on behavior in tail suspension, novel cage, O-maze, food displacement, forced swim, and glucose tolerance tests. **(B)** Study of the effects of 3-week WD feeding of WT, HET, and KO mice on gene expression in the brain and liver.

After a 3-week period of dietary challenge, a cohort of mice was studied, in a novel cage, with O-maze test, depressive-like behaviors in tail suspension and forced swim tests, in the pellet displacement tube (marble) test, a rodent paradigm for a hippocampus-dependent performance (Deacon et al., 2002; Strekalova and Steinbusch, 2010), and a glucose tolerance test (Figure 1A). Another cohort of animals was exposed to the same dietary conditions and sacrificed and dissected for the analysis of gene expression (Figure 1B). During the testing period (days 20 to 23), animals from the WD group received WD and mice from the CD group were fed with standard diet. Six to seven mice per group were used in each study.

### Behavioral Testing

All behavioral tests were carried out during the active period of the animals’ light cycle (09:00–21:00); behavior was recorded and scored offline. The experimenter was blind to the identity of the diet used and the genotype.

#### Novel Cage Test

The novel cage test was performed to assess the exploration of a new environment. Mice were introduced into a standard plastic cage (21 × 27 × 14 cm), under 5-lx lighting. The number of exploratory rears was counted for the first minute of the test by visual observation, as described elsewhere (Costa-Nunes et al., 2015).

#### Elevated O-Maze

The maze consisted of a black circular path (runway width 5.5 cm, diameter 46 cm) that was placed 20 cm above the floor. Illumination intensity was 5 lx. Two opposing compartments were protected by the walls (height 10 cm). Mice were introduced to one of the two closed compartments. Latency to exit to the anxiety-related open compartments of the maze was scored, as described elsewhere (Strekalova et al., 2015).

#### Tail Suspension Test

Mice were subjected to the tail suspension by being hung by their tails with adhesive tape to a rod 50 cm above the floor for 6 min, as described elsewhere (Malatynska et al., 2012). The lighting intensity on the height of the mouse position was 25 lx. The trials were recorded by a video camera positioned directly in front of the mice, while the experimenter observed the session from a distance in a dark area of the experimental room. The latency of the first episode of immobility was scored. In accordance with the commonly accepted criteria of immobility, the immobility behavior was defined as the absence of any movements of the animals’ head and body. The scoring method was previously validated using CleverSys software (CleverSys, Reston, VA, United States) and Noldus software (Wageningen, Netherlands) (Malatynska et al., 2012).

#### Swim Test

This test was carried out as described previously (Strekalova et al., 2015). Mice were placed into a plastic transparent cylinder (Ø 17 cm) filled with water (+23°C, water height 13 cm, and height of cylinder 20 cm) under red lighting. The total duration of floating behavior, defined by the absence of any directed movements of the animals’ head and body, was scored offline during the 6-min period. Latency to float was evaluated as well. The scoring method was previously validated using CleverSys software (CleverSys, Reston, VA, United States) and Noldus software (Wageningen, Netherlands) (Malatynska et al., 2012).

### Pellet Displacement Tube (Marble) Test

All experimental groups were tested for pellet displacement in a tube test as described elsewhere (Deacon et al., 2002; Strekalova and Steinbusch, 2010). A tendency to displace small objects, for example, small stones or food pellets from a tube inside the cage, is species specific in mice and was demonstrated to depend on an intact hippocampal formation (Deacon et al., 2002). Using a paper tube (internal diameter 4 cm, length 10 cm) filled with 20 food pellets and placed in the cage (21 × 27 × 14 cm), the time required for 50% emptying of the tube was recorded.

### Glucose Tolerance Test

Oral glucose tolerance test (OGTT) was performed as described elsewhere (Veniaminova et al., 2017, 2020). The test mice were fasted overnight for 18 h, beginning at 1600. Thereafter, a glucose solution (2 g/kg, 1.8 g/L) was delivered by oral gavage, and blood was sampled from the tail vein. Samples were obtained prior to glucose administration at the time point 0 and 5, 15, 30, 60, 90 min afterward. The level of blood glucose was analyzed using the OneTouch UltraEasy glucometer and strips (LifeScan OneTouch, Dubai, United Arab Emirates). Fasting blood glucose concentrations and the area under the curve (AUC) for the whole test period and for the period between 60 and 90 min after glucose load were analyzed.

### Tissue Dissection

Mice were terminally anesthetized with isoflurane inhalation for a subsequent material collection. The brain of each mouse was perfused with saline and dissected as described elsewhere (Couch et al., 2013). The following brain structures were isolated according to Paxinos and Franklin’s the “Mouse brain in stereotaxic coordinates”: prefrontal cortex (interaural 6.14–5.14), hypothalamus (interaural 3.22–0.88), hippocampus (interaural 2.22–1.00), and dorsal raphe region (interaural -0.56–1.40). Tissue was stored at −80°C until use.

### RNA Extraction and qR T-PCR

Total mRNA was extracted using the RNeasy Mini Kit (Qiagen, Venlo, Netherlands). First-strand cDNA synthesis was performed using High-Capacity cDNA Reverse Transcription Kit (Applied Biosystems, Waltham, MA, United States); 1 μg of total RNA was converted into cDNA. Quantitative PCR for the genes of interest (*Htr1a*, *Htr1b*, *Htr2a*, *Htr2c*, *Htr6*, *Ppargc1a*, *Ppargc1b*, *Tlr4*) and the reference genes (glyceraldehyde 3-phosphate dehydrogenase (*Gapdh*), β-actin (*Actb*), and β-2 microglobulin (*B2m*), was performed using the SYBR Green PCR Master Mix (Applied Biosystems) and QuantStudio 7 Flex Real-Time PCR System (Applied Biosystems). The sequences of primers used are indicated in Supplementary Table S2. The reference genes for normalization were tested for stability using the RefFinder software. The results of the qRT-PCR measurement were expressed as Ct values, and the comparative Ct method was used. Data are given as expression folds compared to the mean expression values in WT mice fed a control diet as described elsewhere (Couch et al., 2013).

### Statistics

Data were analyzed using GraphPad Prism version 8.01 (San Diego, CA, United States). For comparison of the six groups, two-way ANOVA followed by Tukey’s *post hoc* test was used. One-way ANOVA followed by post-test for trend was used for analysis of genotype body weight data at baseline. For comparison of a group mean of 100%, one sample *t* test was performed. Three-way ANOVA analysis was performed using IBM SPSS Statistics 23 (Armonk, NY, United States). The level of significance was set at *p* < 0.05. Data were presented as mean ± SEM or mean.

## Results

### Western Diet and SERT Deficiency Affect Metabolic Parameters

**FIGURE 2:**
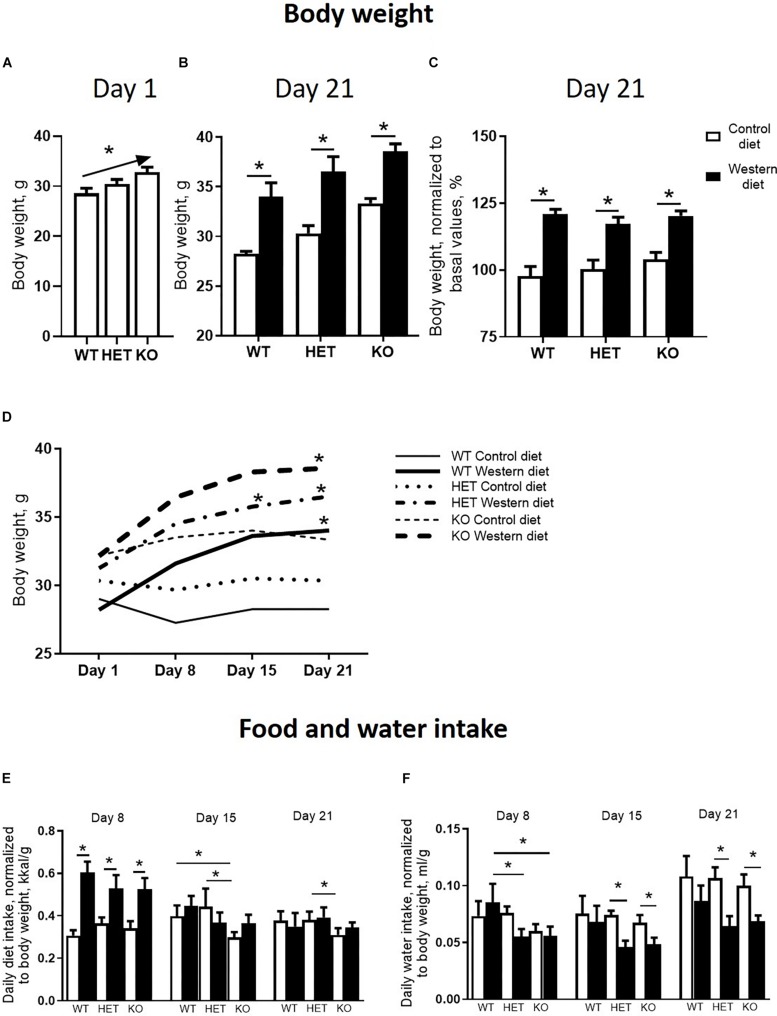
Effects of Western diet feeding on body weight and food and water intake. **(A)** Basal body weight of mice from different serotonin transporter (*Sert*) genotypes: WT, HET, KO. **(B,C)** Body weight after 3-week housing on WD or CD in absolute values and normalized to basal body weight. **(D)** Dynamics of the mouse body weight measured every week during the experiment. Mice fed with WD demonstrated an increase in body weight during the study. **(E,F)** Diet and water intake. *p < 0.05, one-way ANOVA and post-test for trend panel **(A)** or two-way ANOVA and Tukey’s test **(B–F)**, six to seven animals per group were used. Data are shown as mean ± SEM **(A–C,E,F)** or mean **(D)**.

There was a significant difference in body weight at baseline between the *Sert* genotypes (*F* = 4.547, *p* = 0.016, one-way ANOVA). A linear increase in body weight from the WT to KO group (*p* = 0.005, post-test for trend) (Figure 2A) was found. Two-way ANOVA revealed a significant effect of the diet type on the body weight measured both in absolute values and normalized to basal values (*p* < 0.05) (Table 1) after 3 weeks of feeding as measured on day 21. The body weight of WT, HET, and KO mice fed with WD was increased compared with the respective genotype-matched control groups (absolute body weight values: *p* = 0.010, *p* = 0.001, and *p* = 0.005, respectively, Tukey’s test; normalized to basal values: *p* < 0.001, *p* < 0.001, and *p* < 0.001, respectively, Tukey’s test) (Figures 2B,C). A significant interaction between the diet type and the day of the experiment and a significant effect of the genotype were found for body weight changes (*F* = 6.452, *p* < 0.001, and *F* = 28.827, *p* < 0.001, three-way ANOVA). No differences between the groups were found on days 1 and 8. On day 15, body weight was increased in HET-WD mice compared to control HET mice (*p* = 0.014, Tukey’s test) (Figure 2D); on day 21, all *Sert* genotype groups fed with WD displayed increased body weight compared to controls (*p* = 0.044 for WT-WD, *p* = 0.002 for HET-WD, *p* = 0.020 for KO-WD, Tukey’s test). Thus, 3-week WD feeding resulted in body weight increase in all *Sert* genotypes.

**TABLE 1:** Two-way ANOVA results for statistical analysis of metabolic parameters.

Metabolic parameters						

Parameter	Interaction, *F*	Interaction, *p*	Genotype, *F*	Genotype, *p*	Diet, *F*	Diet, *p*
Body weight on D21, absolute values	0.09742	0.9075	8.510	**0.0012**	37.98	**<0.001**
Body weight on D21, normalized to basal values	0.8320	0.4450	0.9820	0.3863	70.65	**<0.001**
Fasting blood glucose level	1.897	0.1710	7.369	**0.0030**	6.514	**0.0172**
Area under the curve (AUC) for glucose tolerance test	1.343	0.2808	1.053	0.3651	9.242	**0.0058**
AUC for glucose tolerance test, 60–90 min	2.392	0.1139	0.3612	0.7007	9.357	**0.006**

**F* and *p* values are shown for interaction between genotype and diet, for genotype effect and for diet effect. *p*-values <0.05 are marked in bold.*

As revealed by the three-way ANOVA, there was a significant interaction between genotype, diet, and experimental day for daily diet calorie intake (*F* = 5.944, *p* < 0.001). During the first week of the experiment, daily calorie intake was increased in WT, HET, and KO mice fed with WD compared to mice fed with CD (*p* < 0.001, *p* < 0.001, and *p* < 0.001, respectively, Tukey’s test) (Figure 2E). Then, during the second week, calorie intake was decreased in KO mice fed with CD compared to WT and HET fed with CD (*p* = 0.027 and *p* = 0.030, respectively, Tukey’s test). During the third week, calorie intake was decreased in KO-CD mice compared to HET-CD (*p* = 0.0231, Tukey’s test). Similar results were obtained for daily diet intake measured in grams per kg of body weight (Supplementary Figure S1). Significant interaction between genotype and diet and experimental day and diet was found for daily water intake (*F* = 15.487, *p* < 0.001, and *F* = 20.020, *p* < 0.001, respectively, three-way ANOVA). During the first week, water intake was decreased in HET-WD and KO-WD mice compared to WT-WD (*p* = 0.016 and *p* = 0.018, respectively, Tukey’s test) (Figure 2F). During the second and third weeks, HET and KO groups fed with WD showed decreased water intake compared to the respective genotype-matched groups fed with CD (HET-WD: *p* < 0.001 and *p* < 0.001, KO-WD: *p* = 0.0003 and *p* < 0.0001, respectively, Tukey’s test). In that way, *Sert* deficiency resulted in a decreased diet intake in KO mice and decreased water intake in HET and KO after WD exposure.

**FIGURE 3:**
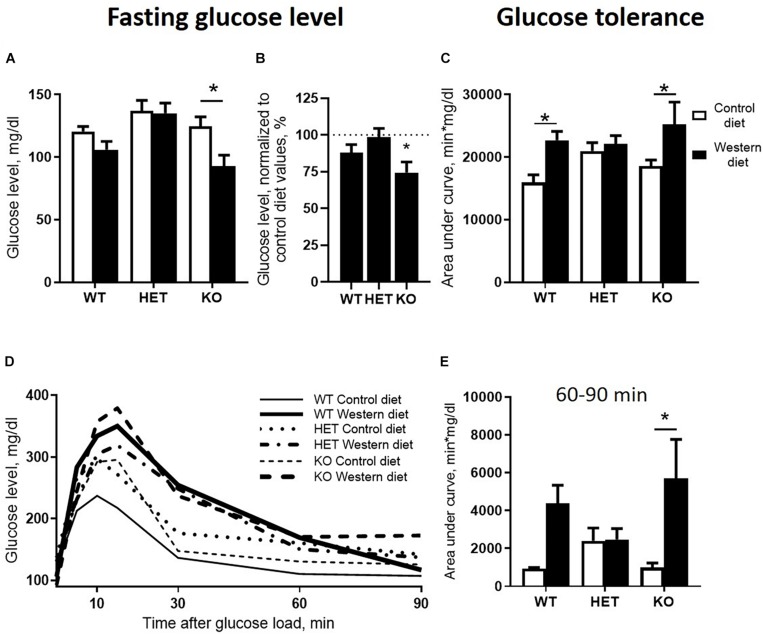
Effects of Western diet feeding on glucose tolerance. **(A)** Fasting blood glucose level after 18 h of food deprivation. WD decreased fasting glucose level in KO mice. **(B)** WD group basal glucose levels normalized to respective genotype group fed with CD values in comparison with 100%. **(C)** Area under glucose tolerance curve. WD increased area under the curve (AUC)_ in WT and KO mice. **(D)** Glucose tolerance curve. **(E)** Area under glucose tolerance curve for the period between 60 and 90 min. *p < 0.05, two-way ANOVA and Tukey’s test **(A,C,E)** or one sample *t* test vs. 100% **(B)**, six to seven animals per group were used. Data are shown as mean ± SEM **(A–C, E)** or mean **(D)**.

We found significant effects of the diet type and the genotype (two-way ANOVA, *p* < 0.05) (Table 1) on the fasting blood glucose levels. Glucose levels after 18 h of food deprivation were decreased in KO mice fed with WD compared to KO fed with CD (*p* = 0.024, Tukey’s test) (Figure 3A). Fasting blood glucose levels normalized to the respective genotype-matched CD group values were also decreased in KO-WD compared to 100% (*t* = 3.528, *p* = 0.0243, one sample *t* test) (Figure 3B). Two-way ANOVA analysis demonstrated a significant diet effect (*p* < 0.05) (Table 1 and Figure 3C) on the area under the curve (AUC) calculated for the glucose tolerance curve (Figure 3D). *Post hoc* analysis revealed a significant increase in AUC in WT and KO mice fed with WD compared to the respective control groups (*p* = 0.021 and *p* = 0.028, respectively, Tukey’s test). No differences in AUC were found between the HET-CD and HET-WD groups. There was a significant diet effect on the AUC calculated for the period between 60 and 90 min after glucose load (*p* < 0.05) (Table 1). This parameter was significantly increased in the KO-WD compared to KO-CD group (*p* = 0.021, Tukey’s test) (Figure 3E). These results indicate that the impairment in glucose tolerance due to feeding with WD was exacerbated in the KO mice in comparison to the WT but was absent in the HET group.

### Effects of Western Diet and SERT Deficiency on the Expression of Markers of Mitochondrial Activity and *Tlr4* in the Brain and Liver

**FIGURE 4:**
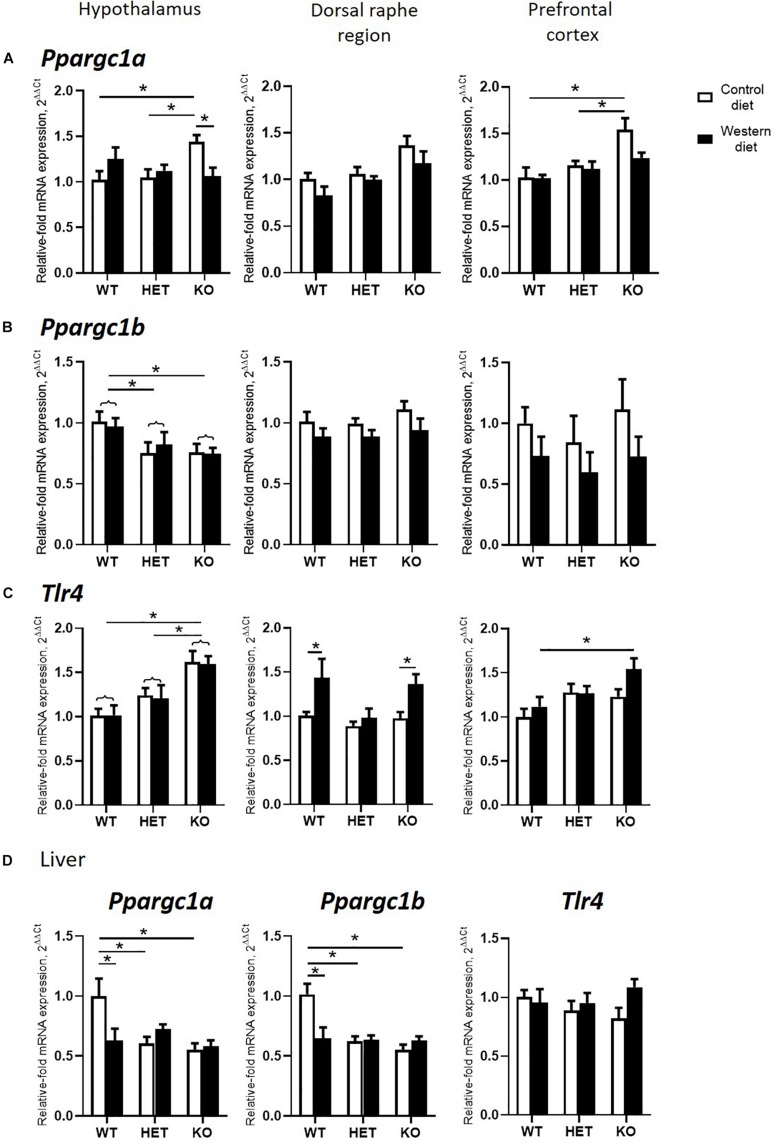
Effects of Western diet and SERT deficiency on brain and liver gene expression of markers of mitochondrial activity and *Tlr4*. **(A–C)** *Ppargc1a*, *Ppargc1b*, and *Tlr4* expression in hypothalamus, dorsal raphe region, and prefrontal cortex. *Tlr4* expression in dorsal raphe region was significantly increased in WT and KO, but not HET mice fed with WD, compared to respective groups fed with CD. **(D)** *Ppargc1a*, *Ppargc1b*, and *Tlr4* expression in the liver. *p < 0.05, two-way ANOVA and Tukey’s test, main genotype effect is marked with braces, six to seven animals per group were used. Data are shown as mean ± SEM.

A significant interaction between the genotype and diet was shown in mRNA levels of *Ppargc1a* in the hypothalamus and hippocampus (two-way ANOVA, *p* < 0.05) (Table 2 and Supplementary Figure S2). The effect of genotype was significant in the dorsal raphe region and prefrontal cortex (*p* < 0.05) (Table 2). *Post hoc* analysis revealed a significant increase in *Ppargc1a* expression levels in the hypothalamus and the prefrontal cortex of KO-CD in comparison to the WT and HET mice fed with CD (*p* = 0.039 and *p* = 0.045 for hypothalamus and *p* = 0.002 and *p* = 0.006 for prefrontal cortex, Tukey’s test) (Figure 4A). *Ppargc1a* expression in the hypothalamus of KO-WD was decreased in comparison to KO-CD (*p* = 0.045, Tukey’s test).

**TABLE 2:** Two-way ANOVA results for statistical analysis of *Ppargc1a* and *b* and *Tlr4* gene expression.

Brain gene expression							

Gene	Brain structure	Interaction, *F*	Interaction, *p*	Genotype, *F*	Genotype, *p*	Diet, *F*	Diet, *p*
Ppargc1a	HT	6.003	**0.0054**	1.799	0.1793	0.1123	0.7394
	DR	0.3308	0.7205	7.713	**0.0017**	3.750	0.0609
	HIP	3.455	**0.0418**	1.745	0.1883	2.065	0.1589
	PF	2.001	0.1492	10.46	**0.0002**	2.956	0.0937
Ppargc1b	HT	0.2827	0.7554	5.436	**0.0085**	0.00613	0.9380
	DR	0.1146	0.8921	0.9017	0.4166	5.301	**0.0284**
	HIP	0.1623	0.8508	2.629	0.0856	0.01598	0.9001
	PF	0.3177	0.7301	0.03676	0.9639	5.088	**0.0313**
Tlr4	HT	0.01194	0.9881	13.47	**<0.0001**	0.04089	0.8409
	DR	2.007	0.1509	4.994	**0.0130**	11.08	**0.0022**
	HIP	0.6018	0.5529	1.317	0.2800	0.2996	0.5874
	PF	1.370	0.2678	5.296	**0.0100**	2.930	0.0961

**Liver gene expression**							

Ppargc1a		6.052	**0.0056**	6.143	**0.0053**	1.733	0.1969
Ppargc1b		9.094	**0.0007**	10.46	**0.0003**	4.501	**0.0412**
Tlr4		1.696	0.1978	0.2381	0.7893	1.668	0.2048

**F* and *p* values are shown for interaction between genotype and diet, for genotype effect, and for diet effect. HT, hypothalamus; DR, dorsal raphe region; HIP, hippocampus; PF, prefrontal cortex. *p*-values <0.05 are marked in bold.*

Two-way ANOVA revealed a significant genotype effect on mRNA levels of *Ppargc1b* in the hypothalamus (*p* < 0.05) (Table 2). In addition, there was a significant diet effect in the dorsal raphe region and prefrontal cortex (*p* < 0.05) (Table 2) on the expression of *Ppargc1b*. No differences were found in the hippocampus (Supplementary Figure S2). *Ppargc1b* expression levels were decreased in the hypothalamus in the KO and HET mice when compared to the WT mice (*p* = 0.0376 and *p* = 0.0095, respectively, Tukey’s test) (Figure 4B).

We found a significant effect of the genotype on mRNA levels of *Tlr4* in the hypothalamus and the prefrontal cortex (two-way ANOVA, *p* < 0.05) (Table 2). Also, there was a significant diet effect in the dorsal raphe region (*p* < 0.05) (Table 2). No differences were found in the hippocampus (Supplementary Figure S2). *Post hoc* analysis showed an increase in *Tlr4* expression levels in the hypothalamus of KO mice compared to WT and HET groups (*p* < 0.001 and *p* = 0.005, respectively, Tukey’s test) (Figure 4C). In addition, a significant increase in *Tlr4* expression was detected in the dorsal raphe region in WT and KO mice fed with WD compared to groups fed with CD (*p* = 0.042 and *p* = 0.040, respectively, Tukey’s test) but not in HET mice, and in the prefrontal cortex in the KO-WD group compared to the WT-WD group (*p* = 0.0403, Tukey’s test). Thus, the most prominent increase in *Tlr4* expression was found in KO fed WD. No effect of WD on *Tlr4* expression was observed in HET mice.

In the liver, there was a significant interaction between genotype and diet on the expression of *Ppargc1a* and *Ppargc1b* (two-way ANOVA, *p* < 0.05) (Table 2). *Ppargc1a* and *Ppargc1b* expression levels in the liver were decreased in WT-WD compared to WT-CD (*p* = 0.026 and *p* = 0.002, respectively, Tukey’s test) (Figure 4D), and in HET and KO mice fed with CD compared to the WT mice fed with CD (*p* = 0.011, *p* = 0.002 and *p* < 0.001, *p* < 0.001, respectively, Tukey’s test). No differences between the groups were found in the *Tlr4* expression in the liver.

### Changes in Brain Expression of Serotonin Receptors by Western Diet and SERT Deficiency

**FIGURE 5:**
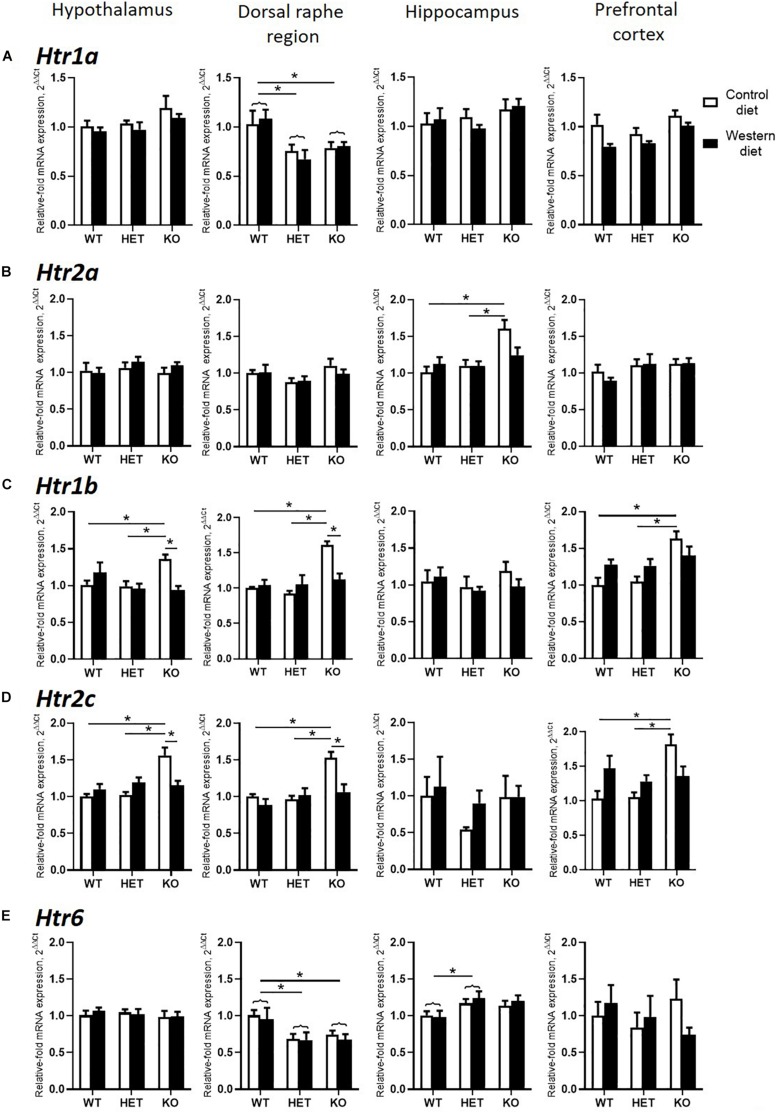
Changes in brain expression of serotonin receptors by Western diet and SERT deficiency. **(A,B)** *Htr1a* and *Htr2a* expression in hypothalamus, dorsal raphe region, hippocampus, and prefrontal cortex. **(C,D)** *Htr1*b and *Htr2c* expression in the brain. KO mice fed with CD compared to WT and HET mice fed with CD displayed significant increase in *Htr1b* and *Htr2c* expression levels in hypothalamus, dorsal raphe region, and prefrontal cortex. Compared to KO mice fed with CD, KO mice fed with WD demonstrated significant decrease in *Htr1b* and *Htr2c* expression levels in hypothalamus and dorsal raphe region. **(E)** *Htr6* expression in hypothalamus, dorsal raphe region, hippocampus, and prefrontal cortex. *p < 0.05, two-way ANOVA and Tukey’s test, main genotype effect is marked with braces, six to seven animals per group were used. Data are shown as mean ± SEM.

Two-way ANOVA analysis demonstrated a significant genotype effect on the *Htr1a* mRNA levels in the dorsal raphe region and prefrontal cortex (*p* < 0.05) (Table 3). In addition, a significant effect of diet was shown in the prefrontal cortex (*p* < 0.05) (Table 3). *Htr1a* expression levels in the dorsal raphe region were decreased in the HET and KO mice compared to the WT mice (*p* < 0.001 and *p* = 0.011, respectively, Tukey’s test) (Figure 5A).

**TABLE 3:** Two-way ANOVA results for statistical analysis of serotonin receptor gene expression.

Brain gene expression							

Gene	Brain structure	Interaction, *F*	Interaction, *p*	Genotype, *F*	Genotype, *p*	Diet, *F*	Diet, *p*
Htr1a	HT	0.07188	0.9308	2.915	0.0664	1.419	0.2409
	DR	0.3828	0.6850	8.898	**0.0008**	0.00130	0.9715
	HIP	0.5541	0.5792	2.066	0.1407	0.02632	0.8720
	PF	0.8123	0.4523	6.690	**0.0035**	9.665	**0.0038**
Htr2a	HT	0.4899	0.6165	0.8927	0.4180	0.8340	0.3669
	DR	0.5235	0.5971	2.932	0.0669	0.2083	0.6510
	HIP	3.485	**0.0408**	9.263	**0.0005**	1.188	0.2827
	PF	0.3957	0.6759	2.289	0.1152	0.2406	0.6266
Htr1b	HT	14.07	**<0.0001**	1.108	0.3407	0.7577	0.3895
	DR	8.429	**0.0010**	13.71	**<0.0001**	2.331	0.1358
	HIP	0.7293	0.4889	0.9170	0.4084	0.4561	0.5035
	PF	4.426	**0.0189**	10.70	**0.0002**	1.309	0.2600
Htr2c	HT	9.803	**0.0004**	10.14	**0.0003**	0.6533	0.4240
	DR	5.308	**0.0097**	10.55	**0.0003**	6.429	**0.0158**
	HIP	0.2895	0.7503	1.057	0.3574	0.6697	0.4183
	PF	6.560	**0.0036**	6.111	**0.0050**	0.4158	0.5229
Htr6	HT	0.2326	0.7936	0.3814	0.6856	0.07229	0.7895
	DR	0.05340	0.9481	5.580	**0.0079**	0.4079	0.5272
	HIP	0.2349	0.7918	4.290	**0.0211**	0.3645	0.5497
	PF	1.420	0.2546	0.2677	0.7666	0.09352	0.7615

**F* and *p* values are shown for interaction between genotype and diet, for genotype effect and for diet effect. HT, hypothalamus; DR, dorsal raphe region; HIP, hippocampus; PF, prefrontal cortex. *p*-values <0.05 are marked in bold.*

There was a significant interaction between the genotype and diet in mRNA levels of *Htr2a* in the hippocampus (two-way ANOVA, *p* < 0.05) (Table 3). *Htr2a* expression was elevated in the hippocampus of the KO-CD mice compared to the WT-CD and HET-CD mice (*p* = 0.001 and *p* = 0.006, Tukey’s test) (Figure 5B).

A significant interaction between genotype and diet in mRNA levels of *Htr1b* receptor was found in the hypothalamus, dorsal raphe region, and prefrontal cortex (two-way ANOVA, *p* < 0.05 (Table 3) but not in hippocampus. *Post hoc* analysis revealed a significant increase in *Htr1b* expression in the hypothalamus, dorsal raphe region, and prefrontal cortex of the KO-CD group compared to the WT-CD and HET-CD groups (*p* = 0.040 and *p* = 0.020 for the hypothalamus, *p* < 0.001 and *p* < 0.001 for the dorsal raphe region, and *p* = 0.001 and *p* = 0.001 for the prefrontal cortex, Tukey’s test) (Figure 5C). In addition, *Htr1b* expression levels were decreased in the hypothalamus and the dorsal raphe region of the KO mice fed with WD in comparison to the KO mice fed with CD (*p* = 0.006 for the hypothalamus, *p* = 0.002 for the dorsal raphe region, Tukey’s test).

We found a significant interaction between genotype and diet in mRNA levels of the *Htr2c* receptor in the hypothalamus, dorsal raphe region, and prefrontal cortex (two-way ANOVA, *p* < 0.05) (Table 3), but not in hippocampus. *Htr2c* expression levels in the hypothalamus, dorsal raphe region, and prefrontal cortex of the KO mice fed with CD were increased compared to the WT and HET mice fed with CD (*p* < 0.001 and *p* < 0.001 for the hypothalamus, *p* = 0.003 and *p* < 0.001 for the dorsal raphe region, and *p* = 0.003 and *p* = 0.002 for the prefrontal cortex, Tukey’s test) (Figure 5D). Decreased *Htr2c* expression levels in the hypothalamus and the dorsal raphe region of KO-WD compared to KO-CD were also detected (*p* = 0.002 for the hypothalamus, *p* = 0.003 for the dorsal raphe region, Tukey’s test).

There was a significant effect of genotype on mRNA levels of *Htr6* receptor in the hippocampus and the dorsal raphe region (*p* < 0.05) (Table 3). In the hippocampus, *Htr6* expression was increased in the HET mice compared to the WT (*p* = 0.029, Tukey’s test) (Figure 5E). *Htr6* expression levels were significantly decreased in the dorsal raphe region of the HET and KO mice compared to the WT mice (*p* = 0.010 and *p* = 0.023, Tukey’s test). In that way, the expression of *Htr1b*, *Htr2a*, and *Htr2c* was increased in the KO group fed with CD. Feeding with WD inversed these changes. *Sert*-deficient mice, irrespective of the diet, demonstrated a decreased *Htr1a* and *Htr6* expression in the dorsal raphe region.

### Western Diet and SERT Deficiency Affected Emotionality and Hippocampus-Dependent Performance

**FIGURE 6:**
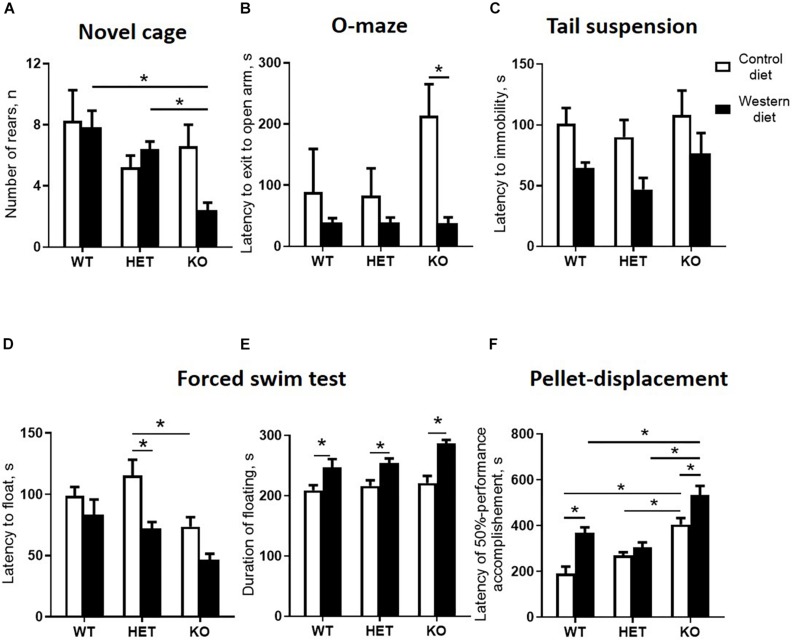
Western diet and SERT deficiency affected behavioral parameters, especially in KO mice. **(A)** Number of rears during the fires minute in novel cage test. KO group fed with WD compared to WT and HET mice fed with WD showed decrease in the number of rears. **(B)** Latency to exit to open arm in O-maze. The latency to exit to open arm was decreased in the KO mice fed with WD compared to the KO group fed with CD. **(C)** Latency to immobility in tail suspension test. **(D)** Latency to floating in swim test. **(E)** Duration of floating in swim test. Compared to groups fed with CD, mice from all Sert genotypes fed with the WD demonstrated increased duration of floating in swim test. **(F)** Latency of 50% performance accomplishment in the pellet-displacement test. WT and KO mice fed with WD in comparison with mice fed with CD demonstrated increased latency of 50% performance accomplishment. This parameter was also significantly increased in KO mice fed with CD and WD compared to respective diet WT and HET groups. *p < 0.05, two-way ANOVA and Tukey’s test, six to seven animals per group were used. Data are shown as mean ± SEM.

A comparison of exploratory rearing activity in the novel cage test during the first minute of the test by two-way ANOVA showed a significant genotype effect on the number of rears (*p* < 0.05) (Table 4). The number of rears was decreased in the WD-fed KO group in comparison to the WD-fed WT and HET mice (*p* = 0.0034 and *p* = 0.0441, respectively, Tukey’s test) (Figure 6A), suggesting a reduced exploration in the KO mice fed with WD.

**TABLE 4:** Two-way ANOVA results for statistical analysis of behavioral parameters.

Behavioral parameters						

Parameter	Interaction, *F*	Interaction, *p*	Genotype, *F*	Genotype, *p*	Diet, *F*	Diet, *p*
Number of rears, 1st min	3.336	0.0502	5.034	**0.0136**	1.680	0.2055
Latency to exit lit arm	1.937	0.1652	1.888	0.1724	8.482	**0.0074**
Latency to float	1.080	0.3556	7.790	**0.0025**	13.34	**0.0013**
Duration of floating	1.303	0.2903	3.514	**0.0459**	33.47	**<0.0001**
Latency to immobility	0.08797	0.9161	1.601	0.2216	10.86	**0.0029**
Latency of 50% accomplishment in tube test	4.210	**0.0192**	29.07	**<0.0001**	27.17	**<0.0001**

**F* and *p* values are shown for interaction between genotype and diet, for genotype effect and for diet effect. *p*-values <0.05 are marked in bold.*

In the O-maze test, there was a significant diet effect (two-way ANOVA, *p* < 0.05) (Table 4) on the latency to exit to open arm. This parameter was decreased in the KO mice fed with WD compared to the KO group fed with CD (*p* = 0.0095, Tukey’s test) (Figure 6B), which could be interpreted as a sign of increased impulsivity in the KO-WD group.

In the tail suspension test, two-way ANOVA revealed a significant diet effect (two-way ANOVA, *p* < 0.05) (Table 4) on the latency to immobility (Figure 6C). While no significant differences were observed between the groups, there was a trend of decreasing latency to immobility in the groups fed with WD compared to the respective genotype-matched groups fed with CD. Significant genotype and diet effects (*p* < 0.05) (Table 4) were found in the latency to float and duration of floating in the swim test. *Post hoc* analysis revealed a significant decrease in the latency to floating in the HET mice fed WD and the KO mice fed CD in comparison to the CD-fed HET group (*p* = 0.0276 and *p* = 0.0356, respectively, Tukey’s test) (Figure 6D). Total duration of floating was increased in the WD-fed WT, HET, and KO groups compared to the CD-fed mice (*p* = 0.049, *p* = 0.030, *p* < 0.001, respectively, Tukey’s test) (Figure 6E). Correlation analysis revealed no significant correlation between mouse body weight and duration of floating, latency to floating, and latency to immobility (*p* > 0.05). This data suggests that WD feeding induces depressive-like behavior in all *Sert* genotypes.

In the pellet-displacement test, there was a significant interaction between the genotype and diet type (*p* < 0.05) (Table 4) in the latency of 50% performance accomplishment. The latency of 50% performance accomplishment was increased in WT-WD and KO-WD mice but not in HET-WD compared to mice fed with CD (*p* < 0.001 and *p* = 0.037, respectively, Tukey’s test) (Figure 6F). In addition, this parameter was increased in the KO group fed with CD compared to the WT-CD and HET-CD (*p* < 0.001 and *p* = 0.001, respectively, Tukey’s test) as well as in the KO group fed with WD compared to the WT-WD and HET-WD (*p* < 0.001 and *p* < 0.001, respectively, Tukey’s test). Results of this test suggest that complete *Sert* deficiency impairs hippocampal-dependent performance. The same effect was observed in WT and KO but not in HET mice fed with WD.

Overall, our data indicate that there is a distinct metabolic, molecular, and behavioral effects of the WD on aged mice with complete versus partial *Sert* inactivation (Table 5), which suggest that *Sert*^+/−^ mice are resilient to several key negative effects of the WD (Table 6).

**TABLE 5:** Effects of Western diet in groups with different *Sert* expression.

Group parameter	WT-WD vs. WT-CD	HET-WD vs. HET-CD	KO-WD vs. KO-CD
**Metabolism**			
Body weight	↑	↑	↑
Food intake, week 1	↑	↑	↑
Water intake, weeks 2 and 3	=	↓	↓
Fasting blood glucose	=	=	↓
Glucose tolerance	↓	=	↓↓
**Liver gene expression**			
*Ppargc1a* and *b*	↓	=	=
**Brain gene expression**			
*Ppargc1a*, HT	=	=	↓
*Tlr4*, DR	↑	=	↑
*Htr1b*, HT and DR	=	=	↓
*Htr2c*, HT and DR	=	=	↓
**Behavior**			
Rearing in novel cage	=	=	↓
Latency to exit open arm in O-maze	=	=	↓
Duration of floating in swim test	↑	↑	↑
Latency to float in swim test	=	↓	=
Latency of 50% performance accomplishment in pellet displacement test	↑	=	↑↑

*Most of the metabolic, molecular, and behavioral changes found in WT-WD mice were exacerbated in KO-WD. Some of the effects of the WD were absent in the HET mice compared to the WT and KO. HT, hypothalamus; DR, dorsal raphe region; HIP, hippocampus; PF, prefrontal cortex. Body weight was analyzed after 3 weeks of WD feeding. Food intake was measured in kcal/g of body weight, water intake—in ml/g of body weight.*

**TABLE 6:** Comparison of HET, KO, and WT mice fed with the control diet.

Group parameter	HET-CD vs. WT-CD	KO-CD vs. WT-CD
**Metabolism**		
Basal body weight	↑	↑↑
Food intake, weeks 2 and 3	=	↓
**Liver gene expression**		
*Ppargc1a* and *b*	↓	↓
**Brain gene expression**		
*Ppargc1a*, HT and PF	=	↑
*Ppargc1b*, HT	↓	↓
*Tlr4*, HT	=	↑
*Htr1a*, DR	↓	↓
*Htr2a*, HIP	=	↑
*Htr1b*, HT, DR and PF	=	↑
*Htr2c*, HT, DR and PF	=	↑
*Htr6*, DR	↓	↓
*Htr6*, HIP	↑	=
**Behavior**		
Latency of 50% performance accomplishment in pellet displacement test	=	↑

*While some metabolic, molecular, and behavioral differences with WT were similar in HET and KO, KO mice demonstrated a more distinguishable phenotype. HT, hypothalamus; DR, dorsal raphe region; HIP, hippocampus; PF, prefrontal cortex. Food intake was measured in kkal/g of body weight.*

## Discussion

Our results reveal that the effects of the WD are, in general, exacerbated in aged *Sert* KO mice in relation to the previously established metabolic, molecular, and behavioral changes. In particular, the key hallmarks of the WD-induced syndrome that were observed in the Sert^−/−^ mice and WT controls included decreased glucose tolerance, brain expression of *Tlr4*, and disrupted hippocampus-dependent performance, but these were not observed in the *Sert*^+/−^ animals. Surprisingly, all the genotypes challenged with WD displayed similar changes in weight gain, depressive-like behavior, and suppressed the expression of *Ppargc1a* and *Ppargc1b* in the liver. Most molecular changes that occurred in the two *Sert*-deficient genotypes developed regardless of diet. These observations will be discussed, in turn, below.

The present study on aged mice has, in general, replicated the principal findings reported for young mice fed the WD, such as impaired glucose tolerance, altered expression of *Tlr4, Ppargc1a*, and *Ppargc1b* (Strekalova et al., 2015, 2016), and signs of emotional and cognitive abnormalities (Strekalova et al., 2015, 2016; Veniaminova et al., 2016, 2017, 2020). In comparison with young mice, aged animals exposed to WD gained weight and exhibited less profound changes in the expression of markers of inflammation and mitochondrial function. This is likely due to age-related alterations in the expression of these genes (Scarpulla, 2002; Letiembre et al., 2007; Burgueño et al., 2013). The aged mice displayed a prolonged increase in intake in the amount of calories after the switch to the highly caloric WD, which lasted for at least one week instead of 3 days in young mice (Strekalova et al., 2015). This is in accordance with the well-described age-related dysregulation of consummatory behavior and metabolic processes (Gill et al., 2015) in the WD model.

The aged *Sert*^−/−^ mice fed a WD displayed greater changes in most of the diet-induced abnormalities that were found in the WT controls, including impairment of glucose tolerance, behavioral despair, suppressed novelty exploration and hippocampus-dependent performance, impulsivity in the O-maze, and brain over-expression of *Tlr4*. At the end of the experiment, in comparison with other genotypes, *Sert*^−/−^ mice displayed a significant decrease in the intake of calories and lowered the blood levels of fasting glucose after being fed the WD. Similar findings were obtained after a 24-week exposure of hamsters to a high-fat diet (Guo et al., 2016) and seemed to be underpinned by the facilitated conversion of blood glucose to lipids, in corollary to the present study, and *Sert*^−/−^ mice revealed an increased glucose absorption in the bowel (Greig et al., 2017). Previous work with WD-fed 6-month-old male *Sert*^−/−^ mice reported the opposite effects (Chen et al., 2012), but this appears to be owing to a sex-related bias (Comhair et al., 2011).

Notably, the finding here that there was reduced energy intake in *Sert*^−/−^ mutants rules out the possibility that their weight gain was due to increased diet intake. Instead, the metabolic changes described in the *Sert*^−/−^ genotype are likely to be caused by functional disturbances in the hypothalamic regulation of intake, which might be related to the overexpression of *Tlr4* in this part of the brain. The activation of TLR4 by long-chain saturated fatty acids (lcSFA) was argued to be a direct trigger of inflammatory mechanisms during excessive consumption of WD-like diets, but lcSFAs are not TLR4 agonists and seem to provide a second hit of activation that is dependent upon prior TLR4 activation. However, lcSFAs, such as palmitate, can induce JNK activation in macrophages and are likely to activate microglia and increase TLR4 expression (Lancaster et al., 2018). Generalized brain overexpression of the TLR4 on the gene and protein levels was shown in the WD-exposed young mice (Strekalova et al., 2015). Pro-inflammatory changes and, particularly, elevated expression of *Tlr4* in the hypothalamus, a primary brain area regulating metabolism (Kahn and Flier, 2000), were shown to affect insulin receptor signaling (Wellen and Hotamisligil, 2003; Benomar et al., 2013; Zhao et al., 2017). Accumulating evidence indicates that neuroinflammatory processes of various causes markedly affects insulin receptor sensitivity (Savage et al., 2001; Olefsky and Glass, 2010). The dysregulation of insulin receptor-mediated signaling can result in the suppression of mitochondrial function and decreased expression of *Ppargca1* and *PPargcb1* that is reported in the present study and elsewhere (Savage et al., 2001; Scarpulla, 2002; Burgueño et al., 2013; Strekalova et al., 2016). The latter results in impaired glucose tolerance, decreased metabolic rate, and obesity (Kahn and Flier, 2000; Wellen and Hotamisligil, 2003). Thus, a close functional relationship between decreased SERT function, pro-inflammatory changes, and insulin resistance is supported by the existing literature (Haub et al., 2010; Pomytkin et al., 2015, 2018).

Naïve *Sert*^−/−^ mice displayed increased brain expression of *Ppargc1a*, *Htr2a*, *Htr1b*, and *Htr2c* that were not found in other genotypes and were “reversed” in WD-fed animals. Given the previously demonstrated association between most of these receptors with obesity and aging (Lee et al., 1998; Meltzer et al., 1998; Simansky and Nicklous, 2002; Nonogaki et al., 2006; Ridderstråle et al., 2006), it seems reasonable to hypothesize that these changes may be adaptive changes, and that the WD disrupts these compensatory changes resulting in aggravation of metabolic and behavioral abnormalities in *Sert*^−/−^ mutants. Expression of several genes were found to be similarly, altered in both *Sert*^−/−^ and *Sert*^+/−^ genotypes regardless of the diet composition. Decreases in expression of *Ppargc1b*, *Htr1a*, and *Htr6* in the brain, and of *Ppargc1a* and *Ppargc1b* in the liver were similar in both genotypes and were not affected by WD, which might indicate ceiling effects of complete or partial Sert deficiency on these receptors. Earlier studies revealed a lowered *Htr1a* receptor expression in the dorsal raphe region of female *Sert*^−/−^ and *Sert*^+/−^ mice that was not found in the hypothalamus or hippocampus (Li et al., 2000). Altered function of 5-HT1A and 5-HT6 are known to underlie depressive-like behaviors (Savitz et al., 2009; Wesołowska, 2010), cognitive (Mitchell and Neumaier, 2005; Ögren et al., 2008) and social (Meneses, 2001; Wang et al., 2013) abnormalities, and may contribute to the behavioral changes observed here. One can suggest that the greater reduction in brain *Ppargc1b* expression in mutants than in controls could be associated with more pronounced obesity in the mutants.

Similar to the changes in the expression of serotonin receptors induced by WD reported here were previously reported. For example, cafeteria and high-energy diets decreased brain expression of *Htr2c* receptor in rats (Lopez-Esparza et al., 2015; Beilharz et al., 2018). Mice exposed to high-fat diet displayed changes in the expression of *Htr2a* in the olfactory nucleus and of *Htr2c* in the medial amygdaloid nucleus (Huang et al., 2004). The 5-HT2C and 5-HT1B receptors were shown to play an inhibitory role in the regulation of calorie intake, while elevated gene expression in the hypothalamus was proposed as a compensatory mechanism of hyperphagia in Ay mice (Nonogaki et al., 2006). The 5-HT1B receptor was found to regulate food intake and insulin receptor sensitivity in mice with genetic inactivation of 5-HT2C receptor (Lee et al., 1998; Simansky and Nicklous, 2002). In keeping with our results, altered expression of 5-HT2C receptor was associated with obesity, abnormal feeding behavior (Heisler et al., 1998), as well as insulin resistance and elevated blood glucose concentrations (Zhou et al., 2007). Finally, 5-HT6 receptor ligands were demonstrated to improve insulin receptor sensitivity and regulate blood insulin, glucose concentrations, mechanisms of satiety, and body weight (Heal et al., 2008).

Remarkably, unlike WT controls and *Sert*^−/−^ mice, *Sert*^+/−^ mice did not exhibit the principal hallmarks of the WD-induced syndrome, such as decreases in glucose tolerance and brain expression of *Tlr4*, and disrupted hippocampus-dependent performance. In contrast to *Sert*^−/−^, they showed no changes in the brain expression of *Ppargc1a*, *Htr2a*, *Htr1b*, and *Htr1c* receptors, decreases in calories and water intake, fasting glucose concentration, as well as novelty hypoexploration and impulsive-like behavior in the elevated O-maze. Thus, *Sert*^+/−^ mice were seemingly quite different from the *Sert*^−/−^ genotype consequences following the WD challenge and exhibited partial “resilience” to its negative effects on the metabolic parameters and associated changes. This is generally in line with previous findings suggesting distinct physiology of two genotypes, as has been shown for the expression of HPA regulatory protein-binding protein 5 (FK506) in the pituitary of mice exposed to early life stress model (van der Doelen et al., 2014) and reduced basal corticosterone plasma levels and improved memory performance in the object recognition test in *Sert*^+/−^ mice (van den Hove et al., 2011).

Relative “resilience” of *Sert*^+/−^ mice to the diet might be viewed as an improved ability to adjust to environmental changes associated with this genotype (Belsky et al., 2009). It is also observed in clinical studies that showed beneficial effects of heterozygosity of SERT, such as higher cognitive function in elderly adults (Fiedorowicz et al., 2007) and other differences (van Dyck et al., 2005; Malmberg et al., 2008) and generally greater fitness in heterozygotes, because they show a broader range of gene expression than both homozygotes (Comings and MacMurray, 2000; Homberg and Lesch, 2011). This phenomenon is also discussed in the framework of heterosis (Sonuga-Barke et al., 2011), or outbreeding enhancement, i.e., the improved or increased function of any biological quality in a hybrid offspring (Shull, 1948).

While our interpretation of the data might be considered speculative, the results encourage further research to explore their contribution of heterosis to the resilience phenotype.

## Conclusion

The comparison of the effects of the WD in *Sert*^−/−^ mice *Sert*^+/−^ reveals an intricate interplay between SERT deficiency and regulation of metabolism during aging. Thus, complete versus partial genetic SERT deficiency in aged mice is associated with distinct metabolic, molecular, and behavioral consequences following the WD challenge. While some diet-induced changes were similar in the KO-WD and HET-WD mice, the latter displayed a “rescued” phenotype in terms of dietary-induced decrease in glucose tolerance, neuroinflammation, and hippocampus-dependent behavior. SERT deficiency was found to enhance inflammatory processes (Haub et al., 2010), and null-mutant Sert mice demonstrate higher susceptibility to the effects of WD on Tlr4 expression.

## Data Availability Statement

The datasets generated for this study are available on request to the corresponding author.

## Ethics Statement

The animal study was reviewed and approved by Local ethics committee of C. Bernard University of Lyon and Local ethics committee of First Moscow State Medical University (#11–18).

## Author Contributions

K-PL and TS conceived the study. TS, AS-B, OK, and DA designed the experiments. EV, SM, TS, and DA carried out the animal experiments. EV performed the molecular analyses. EV, RC, and SM performed the data analysis. IC, AK, DA, and TS supervised the project. IC, OK, K-PL, and TS got the funding. EV and TS wrote the initial draft of the manuscript, and all the other authors RC, IC, AS, SM, OK, AK, DA, and K-PL revised the manuscript.

## Conflict of Interest

The authors declare that the research was conducted in the absence of any commercial or financial relationships that could be construed as a potential conflict of interest.
