# Supplementary material for: Metabolic, Molecular, and Behavioral Effects of Western Diet in Serotonin Transporter-Deficient Mice: Rescue by Heterozygosity?
Source: Front Neurosci. 2020 Feb 18;14:24. doi: 10.3389/fnins.2020.00024 (PMC7041415; doi:10.3389/fnins.2020.00024)
Supplement: Supplementary file 1 [file Data_Sheet_1.docx]

Supplementary Material

# Supplementary Tables

**Supplementary Table S1.** **Composition of diets used in the study.** Compared to control diet, Western diet is characterized by increased content of fat, cholesterol and sucrose and high energy density.

| **Diet** | **Western diet** | | **Control diet** | |
| --- | --- | --- | --- | --- |
| % | gm | *kcal* | gm | *kcal* |
| Protein | 16.8 | *15* | 14.3 | *15* |
| Carbohydrate | 50.5 | *43* | 72.0 | *75* |
| Fat | 21.3 | *42* | 4.3 | *10* |
| Total |  | *100* |  | *100* |
| kcal/gm | 4.61 |  | 3.84 |  |
| **Ingredient** | **gm** | ***kcal*** | **gm** | ***kcal*** |
| Casein, 30 Mesh | 106 | *424* | 106 | *424* |
| L-Cystine | 1.6 | *6.4* | 1.6 | *6.4* |
| Sucrose | 150 | *600* | 150 | *600* |
| Maltodextrin 10 | 100 | *400* | 150 | *600* |
| Corn Starch | 216 | *864* | 481 | *1924* |
| Cellulose, BW200 | 50 | *0* | 50 | *0* |
| **Soybean Oil** | **0** | ***0*** | **25** | ***225*** |
| **Palm Oil** | **185** | ***1665*** | **20** | ***180*** |
| Mineral Mix S10026 | 10 | *0* | 10 | *0* |
| DiCalcium Phosphate | 13 | *0* | 13 | *0* |
| Calcium Carbonate | 5.5 | *0* | 5.5 | *0* |
| Potassium Citrate, 1 H2O | 16.5 | *0* | 16.5 | *0* |
| Vitamin Mix V10001 | 10 | *40* | 10 | *40* |
| Choline Bitartrate | 2 | *0* | 2 | *0* |
| **Cholesterol, NF** | **1.8** | ***0*** | **0** | ***0*** |
| **Total** | **867.45** | ***3999*** | **1040.65** | ***3999*** |

**Supplementary Table S2. Sequences for primers used in qPCR.** Specific primer pairs were used in qPCR for gene expression measurement.

|  |  |  |  |
| --- | --- | --- | --- |
| **Gene** | **Gene ID** | **Forward primer** | **Reverse primer** |
| ***Gapdh*** | 14433 | TGCACCACCAACTGCTTAG | GGATGCAGGGATGATGTTC |
| ***Actb*** | 11461 | GGCTGTATTCCCCTCCATCG | CCAGTTGGTAACAATGCCATGT |
| ***B2m*** | 12010 | QuantiTect Primer Assays Cat. No. QT01149547 (Qiagen, Netherlands) | |
| ***Htr1a*** | 15550 | AACCAGTTTTGTGTCCTCTCA | AGCACCTAAATAATTTTCTTCTC |
| ***Htr1b*** | 15551 | CGCCGACGGCTACATTTAC | TAGCTTCCGGGTCCGATACA |
| ***Htr2a*** | 15558 | CAGGCAAGTCACAGGATAGC | TTAAGCAGAAAGAAAATCCCACA |
| ***Htr2c*** | 15560 | CTAATTGGCCTATTGGTTTGGCA | CGGGAATTGAAACAAGCGTCC |
| ***Htr6*** | 15565 | GCATAGCTCAGGCCGTATGT | CACCACTGTGAGAGGTCCAC |
| ***Tlr4*** | 21898 | CTGGCTAGGACTCTGATCATG | GCATTGGTAGGTAATATTAGGAACTA |
| ***Ppargc1a*** | 19017 | CTCCAGTTCCGGCTCCTC | CCCTGTGCTCTCACGTCTG |
| ***Ppargc1b*** | 170826 | CTCCAGTTCCGGCTCCTC | CCCTGTGCTCTCACGTCTG |

# Supplementary Figures

| **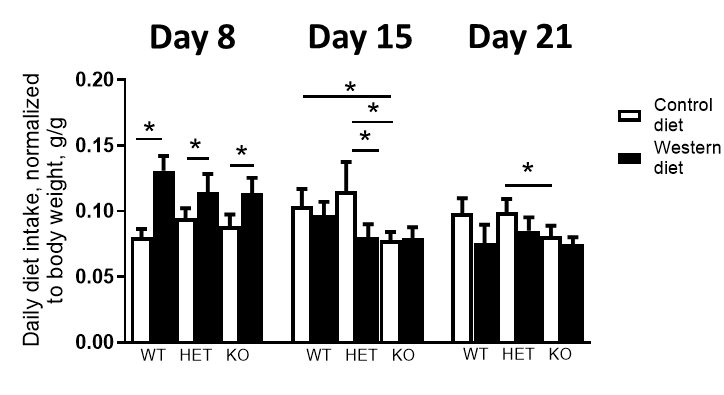** |
| --- |
| **Supplementary Fig. S1. Daily diet intake measured in grams of diet per gram of body weight.** During the 1^st^ week of the experiment, housing on WD led to increased daily diet intake in WT, HET and KO mice compared to mice fed with CD (p<0.001, p=0.044, and p=0.003, respectively). During the 2^nd^ week, diet intake was decreased in KO mice fed with CD compared to WT and HET fed with CD (p=0.027 and p=0.030, respectively) and in HET WD compared to HET CD (p=0.038, Tukey's). During the 3^rd^ week, calorie intake was decreased in KO-CD mice compared to HET-CD (p=0.023, Tukey's test). *p<0.05, two-way ANOVA and Tukey's test, 6-7 animals per group were used. Data are shown as mean ± SEM. |
| **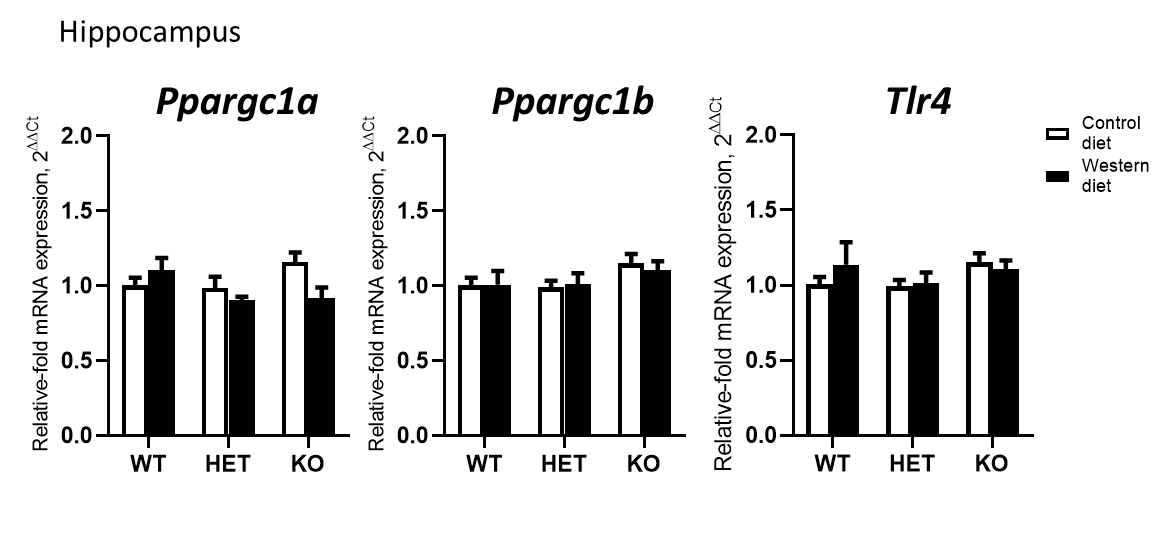** |
| **Supplementary Fig. S2.** ***Ppargc1a,* *Ppargc1b* and *Tlr4* expression in the hippocampus.** No differences in gene expression of *Ppargc1a*, *Ppargc1b* and *Tlr4* in the hippocampus were found between the groups. p>0.05, two-way ANOVA and Tukey's test, 6-7 animals per group were used. Data are shown as mean ± SEM. |
